# The Balance Between the Effectiveness and Safety for Chemotherapy-Induced Nausea and Vomiting of Different Doses of Olanzapine (10 mg Versus 5 mg): A Systematic Review and Meta-Analysis

**DOI:** 10.3389/fonc.2021.705866

**Published:** 2021-09-30

**Authors:** Dong-Yang Wang, Yi Chen, You Zhang, Ying-Qiang Shen

**Affiliations:** State Key Laboratory of Oral Diseases, National Clinical Research Center for Oral Diseases, Chinese Academy of Medical Sciences Research Unit of Oral Medicine of Carcinogenesis and Management, West China Hospital of Stomatology, Sichuan University, Chengdu, China

**Keywords:** olanzapine, vomiting, nausea, meta-analysis, systematic review, antiemetics

## Abstract

**Introduction:**

The aim of this study is to rigorously review the efficacy and safety of olanzapine in chemotherapy-induced nausea and vomiting (CINV) settings including (1) at 5- and 10-mg doses, and (2) the setting of highly emetogenic chemotherapy (HEC) and moderately emetogenic chemotherapy (MEC).

**Methods:**

Embase, Pubmed, and Cochrane Library were searched from the establishment of the database through April 18, 2021. The primary efficacy endpoints were the rate of complete response (CR; no emesis and no rescue), in the acute (0–24 h post-chemotherapy), delayed (24–120 h post-chemotherapy), and overall (0–120 h post-chemotherapy) phases. The secondary efficacy endpoints were the rates of complete control (CC, no nausea, and no emesis), for each phase. Safety endpoints were the rate of somnolence, as assessed by Common Terminology Criteria for Adverse Events (CTCAE) criteria. The Mantel–Haenszel, random, or fixed-effect analysis model was used to compute risk ratios and accompanying 95% confidence intervals for each endpoint. For endpoints that statistically favored one arm, absolute risk differences were computed to assess whether there is a 10% or greater difference, used as the threshold for clinical significance by MASCC/ESMO.

**Result:**

Nine studies reported the use of 10 mg olanzapine to prevent CINV; three studies reported the use of 5 mg olanzapine to prevent CINV. When olanzapine was administered at 10 mg for HEC patients, the six endpoints were statistically and clinically better than the control group. For MEC patients, four out of six endpoints were better than the control group. When olanzapine is administered at 5 mg for MEC patients, four endpoints have statistical and clinical advantages. The sedative effects of 10 and 5 mg olanzapine were statistically more significant than those of the control group. The sedative effect of the 10-mg olanzapine group was more significant than that of the 5-mg olanzapine group, both statistically and clinically.

**Conclusion:**

5 mg olanzapine may be as effective as 10 mg olanzapine for patients with HEC and MEC, and its sedative effect is lower than 10 mg olanzapine. Fewer studies on 5 mg olanzapine have led to uncertain data. In the future, more randomized controlled trials of 5 mg olanzapine are needed to study the balance between the effectiveness and safety of olanzapine.

## 1 Introduction

Chemotherapy-induced nausea and vomiting (CINV) is a common treatment-related side effect that occurs at a rate of approximately 70%–80%. It adversely affects the quality of life of cancer patients and may lead to reduced or stopped chemotherapy (1, 2). Therefore, the prevention and control of CINV is a major clinical problem at present, and it is of clinical significance to study the effectiveness and safety of anti-spitting drugs. CINV can be divided into three phases: acute (the first 24 h after the start of chemotherapy) (3), delay (24 to 120 h after chemotherapy) 4, or overall phase (4)(0 to 120 h after chemotherapy).

The guidelines drawn up by the International Association establish a four-level classification of chemotherapy drugs, consisting of four categories: highly emetogenic chemotherapy (HEC), moderate emetogenic chemotherapy (MEC), low emetogenic chemotherapy, and minimum emetogenic chemotherapy (5). These four categories are based on the percentage of patients with acute vomiting caused by a single drug without anti-spitting prevention. However, the classification does not address the vomiting potential of the combined drug regimen, which is usually determined by the most spitting drug in the combined drug^1^.

The anti-spitting regimen has long been based on a two-drug regimen (including 5-serotonin-3 (5-HT3) receptor antagonists and multi-day dexamethasone) for the prevention of acute and delayed CINV (6). Neuropeptide (NK) 1-receptor antagonists (e.g., aprepitant, rolapitant, and netupitant) are considered promising antiemetics to CINV. Studies reported (7) that the addition of NK-1 receptor antagonists can significantly decrease CINV caused by HEC drugs.

Olanzapine (Olz) is an antipsychotic drug that acts on a variety of receptors. PIRL (8) first reported a case of olanzapine against CINV in 2000. A leukemia patient was in poor mental health due to severe CINV. However, his nausea and vomiting improved significantly after taking 5 mg of olanzapine every night. It is possible to bind to a variety of receptors in the CINV pathway, in particular serotonin receptors (5-HT2A, 5-HT2C, 5-HT3, 5-HT6) and dopamine receptors (D1, D2, D3, and D4) (9) to be non-label antiemetics.

In order to further verify the effect of olanzapine on CINV, Navari et al. (10, 11) conducted two phase II clinical trials in 2005 and 2007. Patients receiving HEC and MEC were given 5-HT3 antagonist and dexamethasone combined with olanzapine to evaluate the antiemetic effect. Another phase II clinical trial (12) showed that olanzapine was more effective in controlling CINV in patients with gynecological tumors in combination with a triple agent. Many Phase II and Phase III trials ^9-11^were conducted on the effectiveness and safety of Olz recently.

A number of systematic reviews and meta-analyses were carried out. However, there is no meta-analysis of the balance between the effectiveness and safety of different doses of olanzapine. This important difference leads to different clinical guidelines recommended.

The Chinese Society of Clinical Oncology (CSCO) recommends olanzapine 5–10 mg and triple solutions to form a vomiting solution for HEC patients, compared to olanzapine and two-joint solution for MEC patients. The National Integrated Cancer Network (NCCN) recommends olanzapine 10 mg and triple regimen as an option for HEC patients (13). American Society of Clinical Oncology (ASCO) guidelines consider olanzapine 5 mg and triple regimen to be safe and effective for HEC patients (14). According to a recent Phase II clinical study (15), 5 mg olanzapine had a better antiemetic effect than 10 mg olanzapine, and its adverse reactions (e.g., sedation) were lower than those of the 10-mg olanzapine group. Therefore, it is necessary to conduct a meta-analysis of the effectiveness and safety of different doses of olanzapine.

In view of the growing interest in olanzapine, a more rigorous review is required. The purpose of this study is to examine the efficacy and safety of Olz in preventing and saving CINV through systematic review and meta-analysis. In addition, in view of the large amount of available data, the purpose of this review is to identify deficiencies in the available literature, providing direction for future research in the CINV environment.

## 2 Method

### 2.1 Inclusion and Exclusion Criteria

The inclusion criteria are as follows: type of study: published randomized controlled trial of olanzapine at different doses in combination with other agents to prevent CINV; subjects: adult cancer patients receiving HEC or MEC chemotherapy; control measures: a general regimen against chemotherapy-induced nausea and vomiting; intervention: olanzapine was added to the standard regimen; evaluation indicators: success rate of prevention of nausea and vomiting, incidence of sedation.

The exclusion criteria are as follows: 1) repeated published literatures (literatures with the most complete and up-to-date data retained); 2) reviews, case reports, meeting abstracts, or trial registration literature; 3) it is not a study on antiemetic chemotherapy in adults or without olanzapine as a variable; 4) full text or incomplete data cannot be obtained.

### 2.2 Literature Retrieval Strategy

A computer search of PubMed, Embase, and Cochrane Library was conducted to collect published randomized controlled trials of olanzapine combined with other drugs at different doses for the prevention of CINV. The relevant literatures were searched from the establishment of the database to April 2021. In order to search the whole literature, we carry out the retrieval mode of subject words + free words, including drug therapy, vomiting or nausea, dexamethasone, olanzapine, and all kinds of free words. See **Appendix 1** for the specific retrieval method.

### 2.3 Literature Screening and Data Extraction

Literature screening, data extraction, and cross-checking were conducted by two researchers independently. In case of disagreement, consensus was reached through discussion or consultation with a third party. Literature screening was conducted by reading the title and abstract and further reading of the full text after excluding the apparently irrelevant literature to determine whether it was included or not.

Data extraction of the main content includes (1) the research of the basic characteristics, including research types, article name, year, authors, published by use of chemotherapy regimens, patients with cancer types, age, gender, patients’ basic information, project for the time range, control group- and experimental group-specific regimen to prevent nausea and vomiting; (2) key elements of bias risk assessment; and (3) outcome indicators and outcome measurement data of concern. The primary efficacy endpoints were the rate of complete responses (CR) in the acute (0–24 h post-chemotherapy), delayed chemotherapy (24–120 h), and overall (0–120 h post-chemotherapy) phases. The secondary efficacy endpoints were the rates of complete control (no nausea and no nausea emesis, CC) for each phase and CR and CC in different chemotherapy regimens. The safety endpoint was the incidence of sedation.

### 2.4 Quality Evaluation of Included Studies

The Cochrane Risk Bias Assessment Tool was used independently by two investigators to assess the risk of bias in the included studies. The method includes 1) random sequence generation (selection bias); 2) allocation concealment (selection bias); 3) blinding of participants and personnel (performance bias); 4) blinding of outcome assessment (detection bias); 5) incomplete outcome data (attrition bias); 6) selective reporting (reporting bias); and 7) other bias. Each item is graded according to “high risk,” “unclear,” and “low risk.”

### 2.5 Statistical Analysis

Meta-analysis of the included studies was performed using RevMan 5.4 software. Relative risk (RR) was used to combine the overall effect for categorical variables. Heterogeneity tests were carried out for the effect sizes of each combined study. If I^2^ > was 50%, indicating heterogeneity, a random-effect model was used, otherwise a fixed-effect model was used. In the overall results, p < 0.05 was considered statistically significant. For endpoints that statistically favored one arm, absolute risk differences were computed to assess whether there is a 10% or greater difference, used as the threshold for clinical significance by MASCC/ESMO.

## 3 Results

### 3.1 Included Studies

From the retrieval policy, 574 records were identified. After deleting 168 duplicate records, a total of 406 records entered the initial screening. Twenty-four studies were obtained by filtering titles and abstract. A second screening of the full text resulted in 12 studies. Twelve studies are reasonably excluded—five studies (16–20) only have Conference abstract, five studies (21–25) do not have specific experimental results, and two studies (26, 27) are unclear in method. A total of 12 studies (28–41) have eligibility criteria for systematic evaluation. All of these studies have been published in peer-reviewed journals. The PRISMA study selection diagram is shown in **Appendix 2**. **Table 1** provides an overview and characteristics of each study, as well as patient characteristics.

**Table 1:** Study demographic.

Reference	Country	Design	Chemotherapy emetogenicity	No. of subjects	Age (mean)		Male, n (%)		Intervention’s additional drug regimens, relative to comparative arm
					Olanzapine	Placebo	Olanzapine	Placebo	
Clemons et al. (28)	Canada	RCT	HEC/MEC	218	50	52	NR	NR	Olanzapine (5 mg oral/day)
Hashimoto et al. (16)	Japan	RCT	MEC	705	65	66	237 (67)	234 (67)	Olanzapine (5 mg oral/day)
Mizukami et al. (30)	Japan	RCT	HEC/MEC	44	63	55	11 (50)	11 (50)	Olanzapine (5 mg oral/day)
Clemmons et al. (39)	Canada	RCT	HEC	101	54	56	29 (57)	31 (62)	Olanzapine (10 mg oral/day)
Vimolchalao et al. (38)	Thailand	RCT	HEC	60	55	53	10 (31.25)	10 (31.25)	Olanzapine (10 mg oral/day)
Jeon et al. (33)	Korea	RCT	MEC	54	60	63	23 (79)	22 (88)	Olanzapine (10 mg oral/day)
Tienchaiananda et al. (37)	Thailand	RCT	HEC	39	49.4	47.3	0	0	Olanzapine (10 mg oral/day)
Mukhopadhyay et al. (34)	India	RCT	HEC/MEC	100	55.04	53.66	27 (54)	31 (62)	Olanzapine (10 mg oral/day)
Navari et al. (31)	America	RCT	HEC	380	56	58	53 (27.6)	52 (27.7)	Olanzapine (10 mg oral/day)
Tan et al. (36)	China	RCT	HEC/MEC	229	M: 54.5 F: 49.58	M: 54 F: 48.25	72 (59.5)	65 (60.2)	Olanzapine (10 mg oral/day)
Yeo et al. (32)	China	RCT	HEC	120	55.5	54.5	0	0	Olanzapine (10 mg oral/day)
Osman et al. (35)	Sudan	PCS	HEC/MEC	131	46	50	14(28)	18(22.2)	Olanzapine (10 mg oral/day)

RCT, randomized controlled trial; HEC, highly emetogenic chemotherapy; MEC, moderately emetogenic chemotherapy; NR, not reported; PO, per os; PCS, prospective comparative study; M, male; F, female.

Eleven studies (28–34, 36–39) are randomized controlled experiments; one study (35)is a prospective control study; and nine studies (28–31, 33, 34, 37–39) compared 5 or 10 mg olanzapine with placebo, while three studies (32, 35, 36) compared the four-drug regimen with standard regimen; and 12 studies differed in the drugs used in the concurrent antiemetic regimens: a corticosteroid, 5-HT3 receptor antagonist, and NK1 receptor antagonist in seven studies (28–32, 36, 39) and a corticosteroid and 5-HT3 receptor antagonist in five studies (33–35, 37, 38).

All studies are CINV for adults. Nine studies (31–39) reported the use of 10 mg olanzapine to prevent CINV; three studies (28–30, 40, 41)reported the use of 5 mg olanzapine to prevent CINV; five studies (31, 32, 37–41) included HEC patients; two studies (29, 33)included MEC patients; and five studies (28, 30, 34–36)included HEC and MEC patients. Of the five studies involving both HEC and MEC, only three studies (34–36) reported the results of HEC and MEC separately, and two studies were not enough for subgroup analysis; eight studies (28, 29, 31–35, 37) reported adverse reactions including sedation, **Table 2** provides the procedures of each study to evaluate the sedative effect of olanzapine; 11 studies (28–34, 36–39) are randomized controlled experiments; 1 study (35) is a prospective control study (40, 41); 9 studies used a double-blind placebo-controlled approach; and 3 studies (32, 35, 36) were unclear on blind methods.

**Table 2:** The procedures to evaluate the sedative effect of olanzapine.

Reference	Assessment procedure
Jeon et al. (33)	Adverse events were graded according to the terminology and grading categories defined in the NCI-CTCAE, ver. 4.03.
Mukhopadhyay et al. (34)	Adverse events were reported by the patients on day 1, on day 3, and on day 8 and also at any time during the study period.
Navari et al. (31)	A study nurse contacted each patient daily on days 2 through 5 to ask about toxic effects. Adverse events were graded according to the terminology and grading categories defined in the NCI-CTCAE, version 4.0.
Osman et al. (35)	Patients were followed up by telephone interview daily from days 1 through 5. The interviewer assessed the severity of sedation, ranging from mild to moderate to severe, reflecting grade 1, grade 2, and grade 3 on CTCAE.
Tienchaiananda et al. (37)	Details of adverse events were collected from all the daily record forms from 4 cycles of AC regimen.
Yeo et al. (32)	Adverse events were graded according to NCI CTCAE v 4.0.
Clemons et al. (28)	The NCI-CTCAE Version 4.02 was used to evaluate the side effects during the Day 2 and 6 telephone calls and at the post chemotherapy clinic visit.
Hashimoto et al., 2019	Adverse events were assessed every 24 h during patients’ hospital admission by clinical study personnel or attending physicians, according to the NCI-CTCAE version 4.0.

NCI-CTCAE, National Cancer Institute’s Common Terminology Criteria for Adverse Events; CTCAE, Common Terminology Criteria for Adverse Events.

All 12 studies reported a CR to antiemetic therapy and defined CR as no vomiting and no rescue; eleven studies (28–30, 32–39) reported a CC to antiemetic therapy and defined CC as no nausea and no emesis. No studies evaluated the bioavailability of the olanzapine in their articles. Only two studies (34, 39) mentioned the plasma half-life of olanzapine in the discussion.

### 3.2 Quality of Included Studies

Each bias risk assessment included in the study is reported in the electronic **Supplementary Material** in **Appendix 3**. Since most studies are double-blind control experiments, the risk of bias in inclusion in the study is low.

### 3.3 Assessment for Publication Bias of Olanzapine for the Prophylaxis of CINV

The funnel diagram is shown in **Appendix 4** electronic **Supplementary Material**.

There is no obvious asymmetry, indicating that there is no obvious publishing bias in this literature.

### 3.4 Efficacy of Olanzapine for the Prophylaxis of CINV

#### 3.4.1 10 mg Olanzapine

##### 3.4.1.1 Complete Response

**Figure 1:**
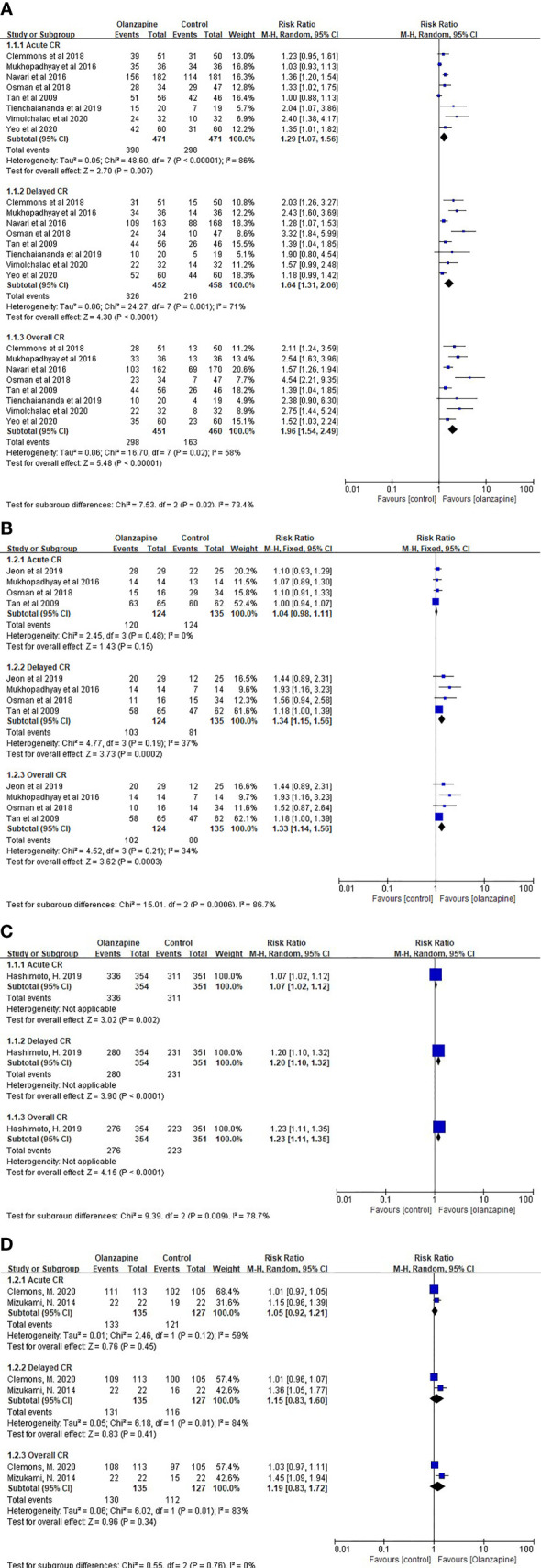
Complete response (CR). **(A)** CR of 10mg Olz for HEC. **(B)** CR of 10mg Olz for MEC. **(C)** CR of 5mg Olz for MEC. **(D)** CR of 5mg Olz for HEC/MEC.

For CR after chemotherapy, 10 mg olanzapine is statistically and clinically superior to the control protocol. For HEC patients, 10 mg Olz has advantages in acute (RR = 1.29, 95% CI = 1.07,1.56), delayed (RR = 1.64, 95% CI = 1.31,2.06), and overall (RR = 1.96, 95% CI = 1.54,2.49) CR (**Figure 1A**). For MEC patients, 10 mg of Olz has advantages in delay (RR = 1.34, 95% CI = 1.15,1.56) and overall (RR = 1.33, 95% CI = 1.14,1.56) CR (**Figure 1B**).

Similarly, for acute (RD = 0.18), delayed (RD = 0.29), and overall (RD = 0.34) CR in HEC patients, as well as delay (RD = 0.21) and overall (RD = 0.20) CR in MEC patients, 10 mg of Olz has a clinical advantage (risk difference greater than 10%, **Table 3**).

**Table 3:** Absolute risk difference between olanzapine and other regimens for statistically significant differences.

Endpoint	Risk difference (95% confidence interval)	Clinically significant?
10 mg OLZ for HEC-acute CR	0.18 [0.07, 0.29]	YES
10 mg OLZ for HEC-delayed CR	0.29 [0.17, 0.40]	YES
10 mg OLZ for HEC-overall CR	0.34 [0.23, 0.44]	YES
10 mg OLZ for MEC-acute CR	0.04 [-0.01, 0.10]	NO
10 mg OLZ for MEC-delayed CR	0.21 [0.11, 0.31]	YES
10 mg OLZ for MEC-overall CR	0.20 [0.10, 0.31]	YES
5 mg OLZ for MEC-acute CR	0.06 [0.02, 0.10]	NO
5 mg OLZ for MEC-delayed CR	0.13 [0.07, 0.20]	YES
5 mg OLZ for MEC-overall CR	0.14 [0.08, 0.21]	YES
5 mg OLZ for HEC/MEC-acute CR	0.05 [-0.07, 0.18]	NO
5 mg OLZ for HEC/MEC-delayed CR	0.13 [-0.14, 0.40]	YES
5 mg OLZ for HEC/MEC-overall CR	0.16 [-0.13, 0.45]	YES
10 mg OLZ for HEC-acute CC	0.16 [0.04, 0.29]	YES
10 mg OLZ for HEC-delayed CC	0.19 [-0.04, 0.41]	YES
10 mg OLZ for HEC-overall CC	0.22 [0.01, 0.44]	YES
10 mg OLZ for MEC-acute CC	0.04 [-0.02, 0.10]	NO
10 mg OLZ for MEC-delayed CC	0.09 [-0.26, 0.43]	NO
10 mg OLZ for MEC-overall CC	0.08 [-0.27, 0.43]	NO
5 mg OLZ for MEC-acute CC	0.06 [0.02. 0.10]	NO
5 mg OLZ for MEC-delayed CC	0.14 [0.08, 0.21]	YES
5 mg OLZ for MEC-overall CC	0.15 [0.09, 0.22]	YES
5 mg OLZ for HEC/MEC-acute CC	0.23 [-0.07, 0.53]	YES
5 mg OLZ for HEC/MEC-delayed CC	0.23 [-0.07,0.53]	YES
5 mg OLZ for HEC/MEC-overall CC	0.22 [0.00, 0.43]	YES
10 mg OLZ-somnolence	0.20 [0.03, 0.37]	YES
5 mg OLZ-somnolence	0.10 [0.04, 0.17]	YES

##### 3.4.1.2 Complete Control

**Figure 2:**
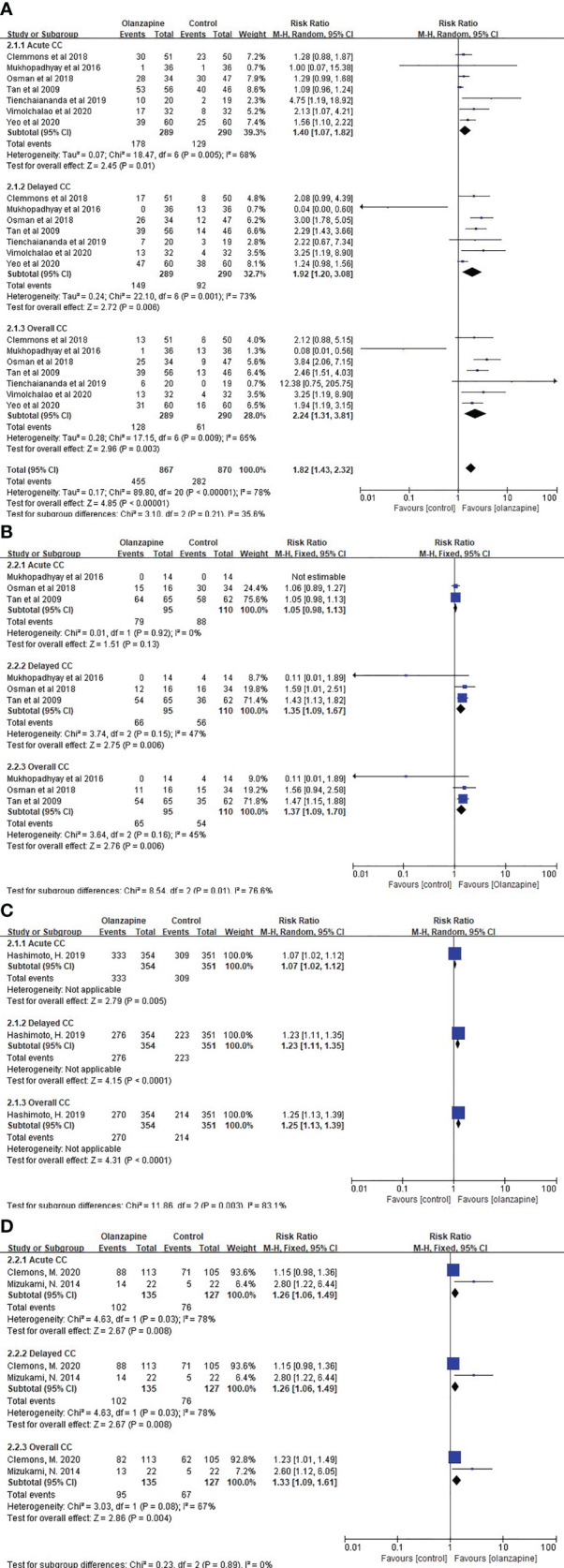
Complete control (CC). **(A)** CC of 10mg Olz for HEC. **(B)** CC of 10mg Olz for MEC. **(C)** CC of 5mg Olz for MEC. **(D)** CC of 5mg Olz for HEC/MEC.

For HEC patients, 10 mg Olz has statistical advantages in acute (RR = 1.40, 95% CI = 1.07,1.82), delayed (RR = 1.92, 95% CI = 1.20,3.08), and overall (RR = 2.24, 95% CI = 1.31,3.81) CC (**Figure 2A**). For MEC patients, 10 mg of Olz has statistical advantages in delayed (RR = 1.35, 95% CI = 1.09,1.67) and overall (RR = 1.37, 95% CI = 1.09,1.70) CC (**Figure 2B**).

Similarly, 10 mg of Olz was superior to the placebo control group clinically only for HEC patients in acute (RD = 0.16), delayed (RD = 0.19), and overall (RD = 0.22) CC (**Table 3**).

#### 3.4.2 5 mg Olanzapine

##### 3.4.2.1 Complete Response

For MEC patients, 5 mg olanzapine has statistical advantages in acute (RR = 1.07, 95% CI = 1.02,1.12), delayed (RR = 1.20, 95% CI = 1.10,1.32), and overall (RR = 1.23, 95% CI = 1.11,1.35) CR (**Figures 1C, D**).

For MEC patients, 5 mg olanzapine was clinically superior to the placebo control group for delay (RD = 0.13) and overall (RD = 0.14) CR. For patients with HEC/MEC, 5 mg olanzapine was clinically superior to the control protocol in delay (RD = 0.13) and overall (RD = 0.16) CR (**Table 3**).

##### Complete Control

For MEC patients, 5 mg olanzapine has statistical advantages in acute (RR = 1.07, 95% CI = 1.02,1.12), delayed (RR = 1.23, 95% CI = 1.11,1.35), and overall (RR = 1.25, 95% CI = 1.13,1.39) CC (**Figure 2C**). Similarly, for patients with HEC/MEC, 5 mg olanzapine has statistical advantages in acute (RR = 1.26, 95% CI = 1.06,1.49), delayed (RR = 1.26, 95% CI = 1.06,1.49), and overall (RR = 1.33, 95% CI = 1.09,1.61) CC (**Figure 2D**).

For MEC patients, 5 mg olanzapine has clinical advantages for delay (RD = 0.14) and overall (RD = 0.15) CC. For patients with HEC/MEC, 5 mg olanzapine was clinically superior to the control protocol in acute (RD = 0.23), delayed (RD = 0.23), and overall (RD = 0.23) CC (**Table 3**).

### 3.5 Safety of Olanzapine for the Prophylaxis of CINV

#### Somnolence

**Figure 3:**
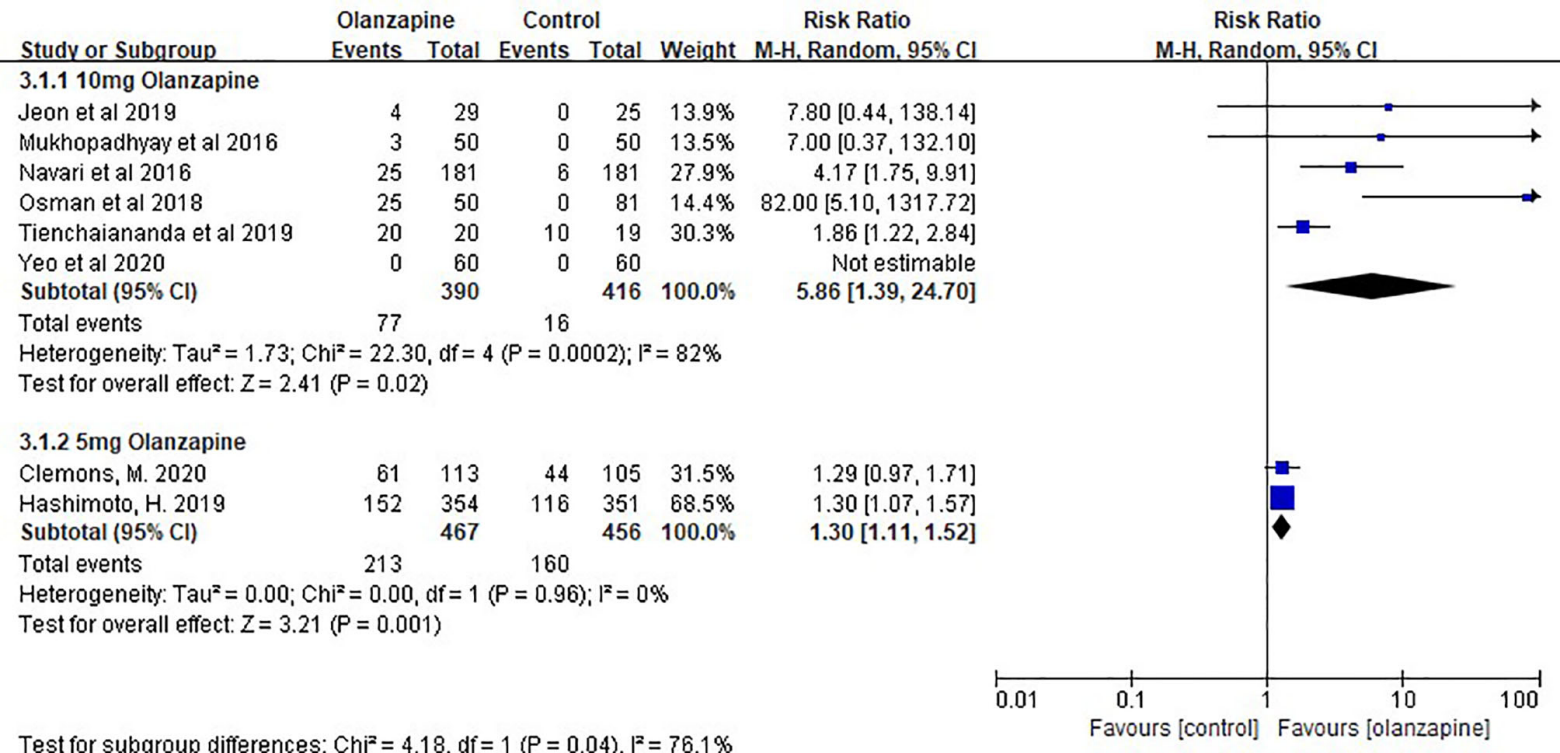
The somnolence effect of Olz(10mg and 5mg).

The sedation of the 10- and 5-mg olanzapine groups was statistically significant than that of the control group. The sedation of the 10-mg olanzapine group was more significant than that of the 5-mg olanzapine group, both statistically (**Figure 3**) and clinically (**Table 3**).

### 3.6 Subgroup Analysis of Different Regions

**Figure 4:**
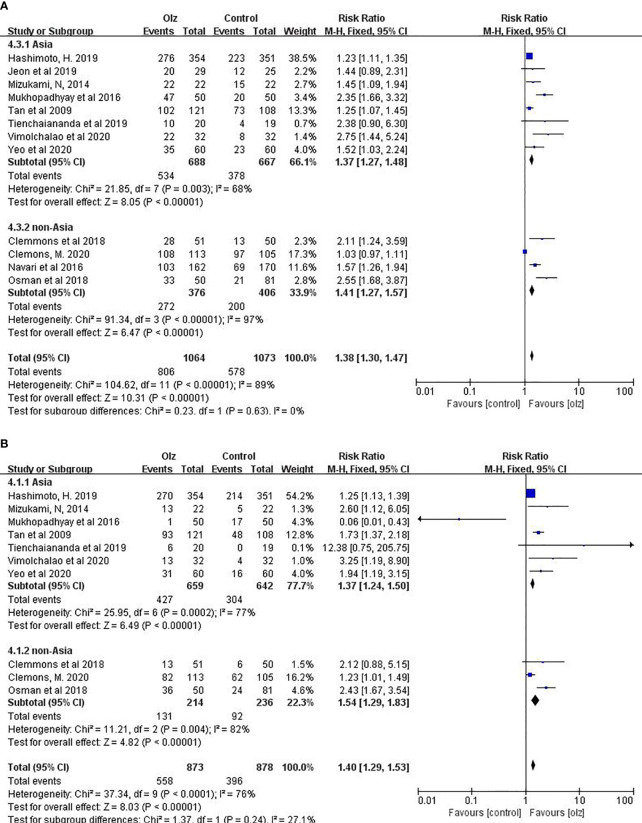
Subgroup analysis of different regions. **(A)** CR of Olz for Asia or non-Asia countries. **(B)** CR of Olz for Asia or non-Asia countries.

Taking into account the ethnic differences in different regions, we conducted a subgroup analysis between the Asian and non-Asian countries. For antiemetic effects of olanzapine, there was no significant difference between Asian and non-Asian countries statistically (**Figure 4**).

## 4 Discussion

Twelve studies were included in this study to explore the balance between the efficacy and sedation of olanzapine in the prevention of CINV, to minimize sedation while ensuring the maximum efficacy, and to provide some guidance for improving the quality of life of patients in terms of medication.

Meta-analysis showed that for adult patients receiving HEC, 10 mg olanzapine was more statistically superior (six endpoints) than 5 mg olanzapine (three endpoints), but their clinical advantages were similar. This result suggests that 10 mg olanzapine may be a prior choice for HEC patients, and 5 mg olanzapine may be a clinical choice for patients without obvious risk of CINV (**Tables 4**, **5**).

**Table 4:** Statistical advantages of olanzapine for the prophylaxis of CINV.

The dose of olanzapine	The category of chemotherapy	CR			CC		
		Acute	Delay	Overall	Acute	Delay	Overall
10 mg	HEC	√	√	√	√	√	√
	MEC		√	√		√	√
5 mg	HEC				√	√	√
	MEC	√	√	√	√	√	√

**Table 5:** Clinical advantages of olanzapine for the prophylaxis of CINV.

The dose of olanzapine	The category of chemotherapy	CR			CC		
		Acute	Delay	Overall	Acute	Delay	Overall
10 mg	HEC	√	√	√	√	√	√
	MEC		√	√			
5 mg	HEC		√	√	√	√	√
	MEC		√	√		√	√

In adult patients receiving MEC, 5 mg olanzapine showed obvious advantage both statistically (six endpoints) and clinically (four endpoints) compared with 10 mg olanzapine (four endpoints and two endpoints). Moreover, 5 mg olanzapine has statistical advantages in acute CR and CC, which suggests the doctors do not need to rescue the patients after the chemotherapy. Therefore, 5 mg olanzapine may be a prior choice for MEC patients.

Compared with the onset of acute nausea and vomiting, both 10 mg olanzapine and 5 mg olanzapine showed better preventive effects in the delayed period and overall. The better effect of olanzapine in the delayed phase and overall may be due to the lower incidence of vomiting and nausea in the acute phase (42).

It is worth paying attention the side effects of olanzapine, which was originally used as an antipsychotic. In the study by Jeon (33), 10 mg olanzapine showed only a few cases of mild sedation in the description of sedation, suggesting that sedation was not significant. However, in the study by Navari (31), a large number of statistical data indicated that the drowsiness effect of 10 mg olanzapine was significantly different from that of placebo. Similar reports have been reported for 5 mg olanzapine, although less than for 10 mg.

Therefore, we also analyzed the sedative effect of two doses of olanzapine in this meta-analysis, and the results showed that there was indeed a sedative effect of olanzapine, but the effect intensity in the 5-mg group was significantly lower than that in the 10-mg group and was not as significant as that in the placebo group. Combined with the above 5-mg olanzapine group in patients receiving MEC, the combination of 5 mg olanzapine may be a better option for the prevention of CINV, but we need to wait for the performance of 5 mg olanzapine in patients receiving HEC and more information on the sedative effect of 5 mg olanzapine.

A recent Phase II clinical study (15) reported that 5 mg olanzapine had a better antiemetic effect than 10 mg olanzapine, and its adverse reactions (e.g., sedation) were lower than those of the 10-mg olanzapine group. As the efficacy of olanzapine in emesis control significantly exceeded that of the placebo, and taking the possible increased risk of adverse drug reactions (ADRs) with the dose increase into consideration, olanzapine at a dose of 5 mg per day should be recommended for the prophylaxis of CINV. If patients fail to achieve CR, the dose can be increased to 10 mg in the next chemotherapy cycle.

At present, the occurrence of CINV still cannot be completely controlled. Multiple studies have shown that the overall response rates of acute and delayed CINV in HEC patients treated with the triple regimen are 75% to 80% and 57% to 70%, respectively, and the incidence of nausea is more than 50%. This still falls short of the expected goal of 100% mitigation of CINV (43–45). The mechanism of action of olanzapine is that it binds to the dopamine receptor, cholinergic receptor, and histamine H1 receptor and plays an antagonistic role. It reduces the stimulation of the chemoreceptor triggering area (CTZ) and thus reduces the occurrence of vomiting. At present, the effect of olanzapine has also been affirmed in the Guidelines for the Prevention and Treatment of Nausea and Vomiting Related to CSCO Anti-tumor Therapy. However, OLZ is currently mainly used in patients who are resistant to the combination of drugs, which may be due to the sedative effect of olanzapine. Our study showed that a low dose of olanzapine may have significant effects in the prevention of CINV and few adverse reactions, which may be helpful for the clinical use of olanzapine after the chemotherapy.

Limitations: (1) Although the drug regimens for the prevention of CINV in the control group included in the experiment all adopted the regimens in the guidelines of various countries, there were differences in the regimens of each trial. There was some bias in remission rate. (2) The cancer type, malignancy degree, and purpose of chemotherapy of cancer patients are different, and some may affect the occurrence of CINV. (3) There are great differences in the descriptions of sedation in the literature, and there is no unified standard to judge the intensity of sedation, which may lead to errors in the results of sedation. (4) The quality of the literature included in this study is different, and the difference in the experimental results may lead to deviation in the results of this meta-analysis. (5) The sample size of some studies is small and may not be representative.

## Data Availability Statement

The original contributions presented in the study are included in the article/**Supplementary Material**. Further inquiries can be directed to the corresponding author.

## Author Contributions

D-YW: data curation, methodology, visualization, writing—original draft, and writing—review and editing. YC: data curation, methodology, visualization, writing—original draft, and writing—review and editing. YZ: conceptualization, methodology, project administration, writing—original draft, and writing—review and editing. Y-QS: conceptualization, methodology, project administration, writing—original draft, and writing—review and editing. All authors contributed to the article and approved the submitted version.

## Funding

This study was supported by the Innovation and Entrepreneurship Training Scheme for university students Program (No. C202110611401) from West China School/Hospital of Stomatology Sichuan University.

## Conflict of Interest

The authors declare that the research was conducted in the absence of any commercial or financial relationships that could be construed as a potential conflict of interest.

## Publisher’s Note

All claims expressed in this article are solely those of the authors and do not necessarily represent those of their affiliated organizations, or those of the publisher, the editors and the reviewers. Any product that may be evaluated in this article, or claim that may be made by its manufacturer, is not guaranteed or endorsed by the publisher.
