# Supplementary material for: The Balance Between the Effectiveness and Safety for Chemotherapy-Induced Nausea and Vomiting of Different Doses of Olanzapine (10 mg Versus 5 mg): A Systematic Review and Meta-Analysis
Source: Front Oncol. 2021 Sep 30;11:705866. doi: 10.3389/fonc.2021.705866 (PMC8514875; doi:10.3389/fonc.2021.705866)
Supplement: Supplementary file 1 [file DataSheet_1.docx]

Appendix:

**1：specific retrieval method.**

**Embase**


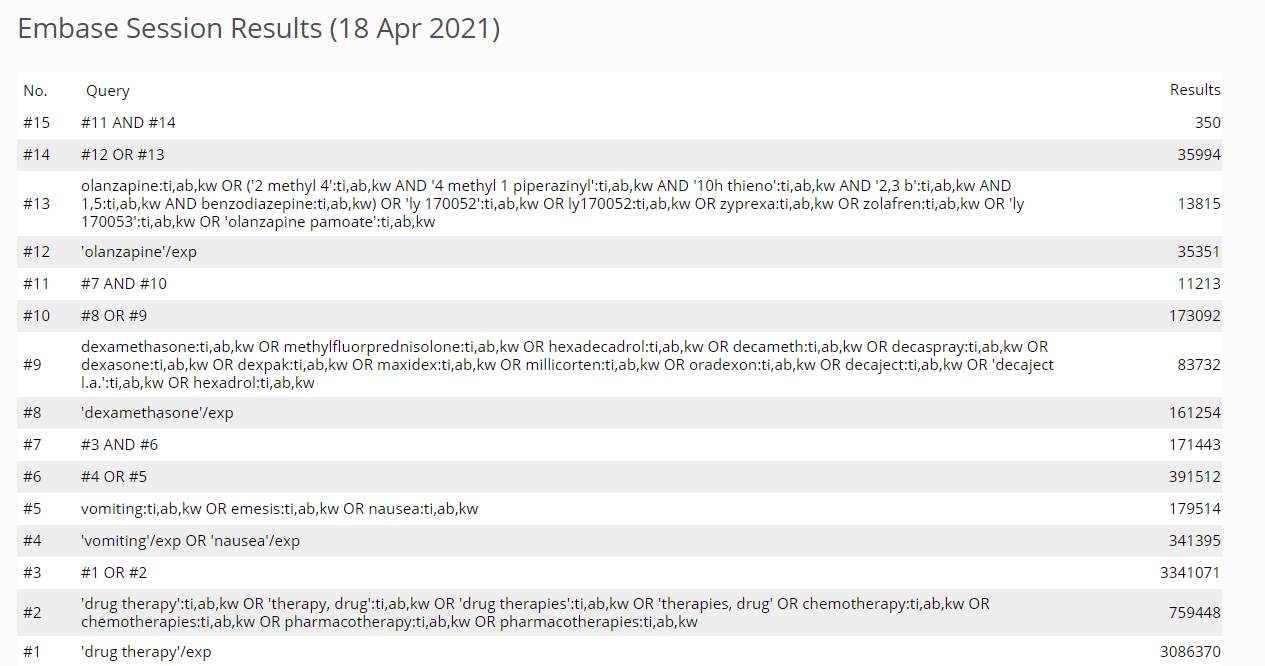


**Pubmed**

Query:

(((((((((((drug therapy[MeSH Terms]) OR (drug therapy[Title/Abstract])) OR (Therapy, Drug[Title/Abstract])) OR (Drug Therapies[Title/Abstract])) OR (Therapies, Drug[Title/Abstract])) OR (Chemotherapy[Title/Abstract])) OR (Chemotherapies[Title/Abstract])) OR (Pharmacotherapy[Title/Abstract])) OR (Pharmacotherapies[Title/Abstract])) AND (((((Vomiting[MeSH Terms]) OR (Vomiting[Title/Abstract])) OR (Emesis[Title/Abstract])) OR (nausea[MeSH Terms])) OR (nausea[Title/Abstract]))) AND ((((((((((((((((Dexamethasone[MeSH Terms]) OR (Dexamethasone[Title/Abstract])) OR (Methylfluorprednisolone[Title/Abstract])) OR (Hexadecadrol[Title/Abstract])) OR (Decameth[Title/Abstract])) OR (Decaspray[Title/Abstract])) OR (Dexasone[Title/Abstract])) OR (Dexpak[Title/Abstract])) OR (Maxidex[Title/Abstract])) OR (Millicorten[Title/Abstract])) OR (Oradexon[Title/Abstract])) OR (Decaject[Title/Abstract])) OR (Decaject-L.A.[Title/Abstract])) OR (Decaject L.A.[Title/Abstract])) OR (Hexadrol[Title/Abstract])) OR ((standard antiemetic therapy[Title/Abstract]) OR (triplet regimen[Title/Abstract])))) AND ((((((((((olanzapine[MeSH Terms]) OR (olanzapine[Title/Abstract])) OR (2-Methyl-4-(4-methyl-1-piperazinyl)-10H-thieno(2,3-b)(1,5)benzodiazepine[Title/Abstract])) OR (LY-170052[Title/Abstract])) OR (LY 170052[Title/Abstract])) OR (LY170052[Title/Abstract])) OR (Zyprexa[Title/Abstract])) OR (Zolafren[Title/Abstract])) OR (LY 170053[Title/Abstract])) OR (Olanzapine Pamoate[Title/Abstract]))

Details:

("drug therapy"[MeSH Terms] OR "drug therapy"[Title/Abstract] OR "therapy drug"[Title/Abstract] OR "drug therapies"[Title/Abstract] OR "therapies drug"[Title/Abstract] OR "Chemotherapy"[Title/Abstract] OR "Chemotherapies"[Title/Abstract] OR "Pharmacotherapy"[Title/Abstract] OR "Pharmacotherapies"[Title/Abstract])

AND ("Vomiting"[MeSH Terms] OR "Vomiting"[Title/Abstract] OR "Emesis"[Title/Abstract] OR "nausea"[MeSH Terms] OR "nausea"[Title/Abstract])

AND ("Dexamethasone"[MeSH Terms] OR "Dexamethasone"[Title/Abstract] OR "Methylfluorprednisolone"[Title/Abstract] OR "Hexadecadrol"[Title/Abstract] OR "Decameth"[Title/Abstract] OR "Decaspray"[Title/Abstract] OR "Dexasone"[Title/Abstract] OR "Dexpak"[Title/Abstract] OR "Maxidex"[Title/Abstract] OR "Millicorten"[Title/Abstract] OR "Oradexon"[Title/Abstract] OR "Decaject"[Title/Abstract] OR "decaject l a"[Title/Abstract] OR "decaject l a"[Title/Abstract] OR "Hexadrol"[Title/Abstract] OR ("standard antiemetic therapy"[Title/Abstract] OR "triplet regimen"[Title/Abstract]))

AND ("Olanzapine"[MeSH Terms] OR "Olanzapine"[Title/Abstract] OR ((((("2 methyl 4"[All Fields] AND "4-methyl-1-piperazinyl"[All Fields]) AND "10h thieno"[All Fields]) AND ("2"[All Fields] AND "3-b"[All Fields])) AND 1,5[UID]) AND "benzodiazepine"[Title/Abstract]) OR ("LY"[All Fields] AND "170052"[Title/Abstract]) OR "Zyprexa"[Title/Abstract] OR "Zolafren"[Title/Abstract] OR "ly 170053"[Title/Abstract] OR "olanzapine pamoate"[Title/Abstract])

Results: 97

**Corchrane**


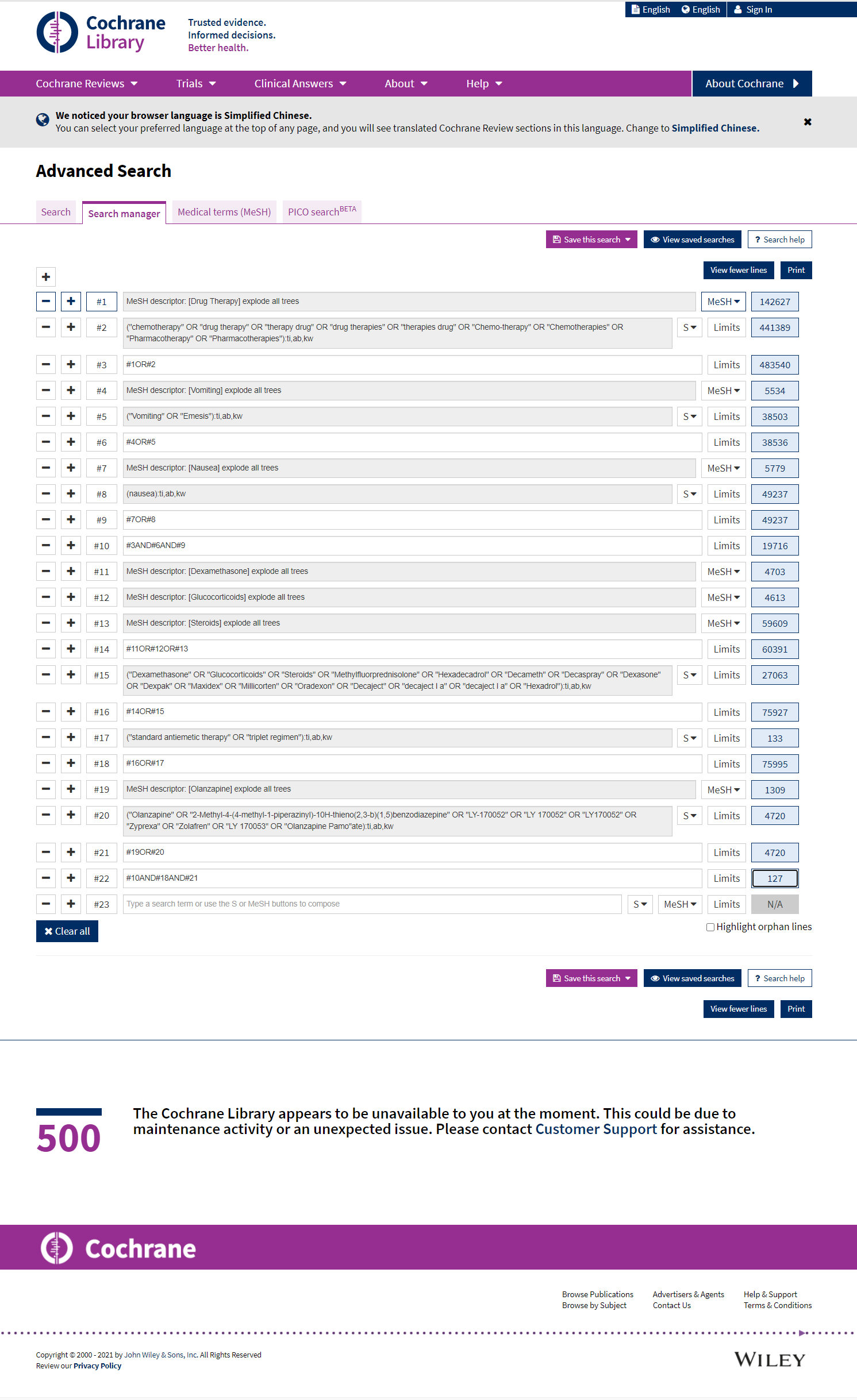


**2：PRISMA:**

Studies in Embase

（n=350）

Studies in Cochrane

（n=127）

Studies in Pubmed

（n=97）

406 records screened after duplicates removed (n=168)

12 full-text articles excluded:

No experimental results: 5 studies

Only the abstract of the meeting: 5 studies

Not randomized controlled trial: 2 studies

24 full-text articles assessed for eligibility

12 randomized controlled trials included in quantitative synthesis (meta-analysis)

Ineligible (n=382)

Non-experimental studies: 185 studies

Not research in CINV for adults:39 studies

Not variable by olanzapine’ dose: 101studies

Can’t get the full text: 41 studies

Test drug other than olanzapine: 16studies

3、**Quality of included studies**

**Risk of bias summary：**

**
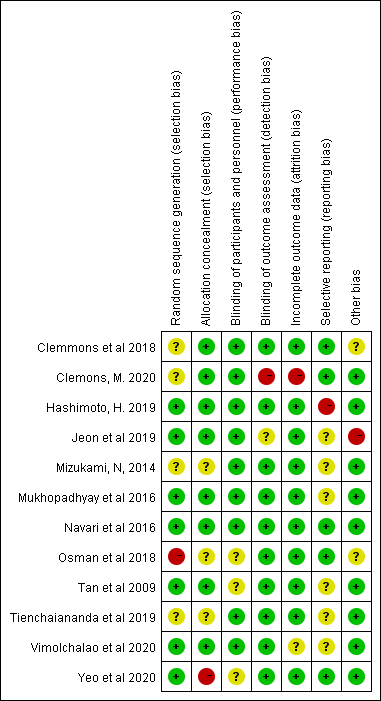
**

**Risk of bias graph**
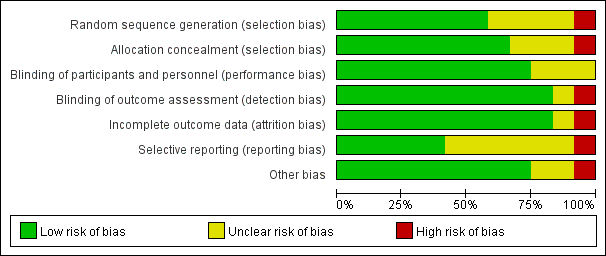


4、**Assessment for publication bias of olanzapine for the prophylaxis of CINV**
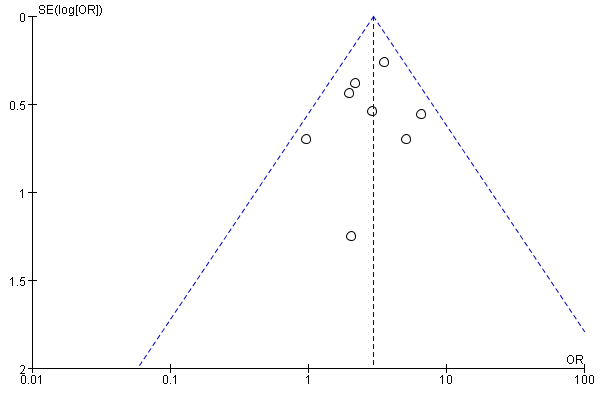
 Funnel plot of comparison: 1 Acute CR, outcome: 1.1 10mg for HEC.


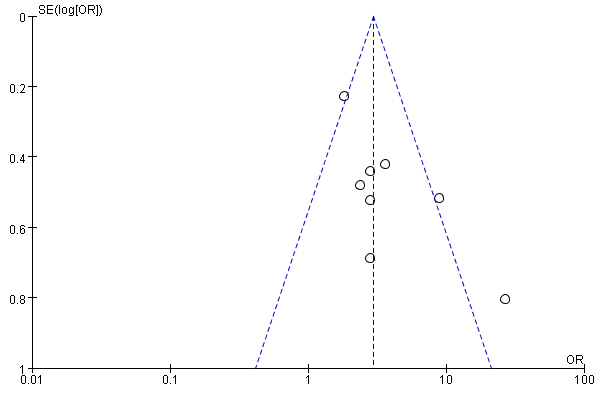
 Funnel plot of comparison: 1 Delayed CR, outcome1.2: 10mg for HEC.


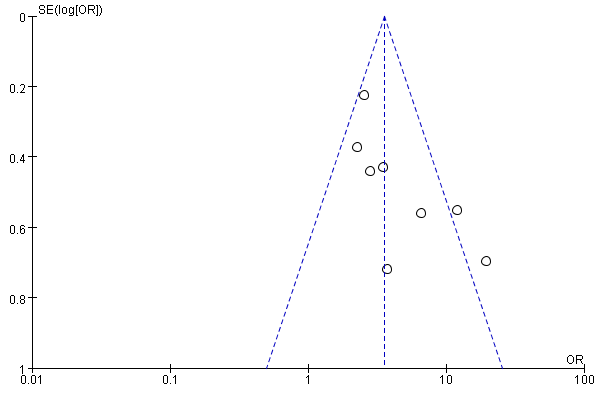
 Funnel plot of comparison: 1 Overall CR, outcome: 1.3 10mg for HEC.
